# Postoperative Admission to a Dedicated Geriatric Unit Decreases Mortality in Elderly Patients with Hip Fracture

**DOI:** 10.1371/journal.pone.0083795

**Published:** 2014-01-15

**Authors:** Jacques Boddaert, Judith Cohen-Bittan, Frédéric Khiami, Yannick Le Manach, Mathieu Raux, Jean-Yves Beinis, Marc Verny, Bruno Riou

**Affiliations:** 1 Université Pierre et Marie Curie (UMRS 956, UMRS 1158), Paris, France; 2 Department of Geriatrics, Groupe hospitalier (GH) Pitié-Salpêtrière, Assistance Publique Hôpitaux de Paris (APHP), Paris, France; 3 Department of Orthopedic Surgery and Trauma, GH Pitié-Salpêtrière, APHP, Paris, France; 4 Departments of Anesthesia & Clinical Epidemiology and Biostatistics, Michael DeGroote School of Medicine, Faculty of Health Sciences, McMaster University, Hamilton, Ontario, Canada; 5 Department of Anesthesiology and Critical Care, GH Pitié-Salpêtrière, APHP, Paris, France; 6 Department of Rehabilitation, Groupe Hospitalier Charles Foix, APHP, Ivry-sur-Seine, France; 7 Department of Emergency Medicine and Surgery, GH Pitié-Salpêtrière, APHP, Paris, France; 8 Institut national de la santé et de la recherche médicale (UMRS 956, UMRS 1158, UMR 689), Paris, France; University of South Florida, United States of America

## Abstract

**Background:**

Elderly patients with hip fracture have a 5 to 8 fold increased risk of death during the months following surgery. We tested the hypothesis that early geriatric management of these patients focused on co-morbidities and rehabilitation improved long term mortality.

**Methods and Findings:**

In a cohort study over a 6 year period, we compared patients aged >70 years with hip fracture admitted to orthopedic versus geriatric departments in a time series analysis corresponding to the creation of a dedicated geriatric unit. Co-morbidities were assessed using the Cumulative Illness Rating Scale (CIRS). Each cohort was compared to matched cohorts extracted from a national registry (n = 51,275) to validate the observed results. Main outcome measure was 6-month mortality. We included 131 patients in the orthopedic cohort and 203 in the geriatric cohort. Co-morbidities were more frequent in the geriatric cohort (median CIRS: 8 vs 5, *P*<0.001). In the geriatric cohort, the proportion of patients who never walked again decreased (6% versus 22%, *P*<0.001). At 6 months, re-admission (14% versus 29%, P = 0.007) and mortality (15% versus 24%, *P* = 0.04) were decreased. When co-morbidities were taken into account, the risk ratio of death at 6 months was reduced (0·43, 95%CI 0·25 to 0·73, *P* = 0.002). Using matched cohorts, the average treatment effects on the treated associated to early geriatric management indicated a reduction in hospital mortality (−63%; 95% CI: −92% to −6%, *P* = 0.006).

**Conclusions:**

Early admission to a dedicated geriatric unit improved 6-month mortality and morbidity in elderly patients with hip fracture.

## Introduction

Worldwide 1.6 million patients suffer a hip fracture each year [1] and as the population continues to age this figure has increased by 25% each decade [2]. In the elderly patient hip fracture has devastating consequences. The hospital mortality of the condition ranges from 2.3% to 13.9% [3]–[7], with patients discharged home having a 5 to 8 fold increased risk of death in the months immediately following surgery [8]. This risk persists well beyond the immediate surgical period with 6 month mortality rates ranging from 12 to 23 % [7], [9]–[12], and it is estimated that hip fractures account for more than 1.5% of all deaths in patients aged 50 years or more [13].

When compared to elective total hip replacements, patients presenting with hip fracture have a 6 to 15 fold mortality risk [14]. This can largely be explained by the high prevalence of pre-existing medical conditions seen in this population: 75% of patients are older than 70 years [1], and 95% of them present with at least one major preoperative comorbidity [15]. However studies suggest that only 1 in 4 of hip fracture associated deaths may be causally related to the fracture itself rather than due to pre-existing medical conditions [13]. This suggests that the insult of the hip fracture destabilizes an elderly population with a high burden of pre-existing morbidities thereby resulting in excess mortality.

Despite the magnitude of this problem there are no established effective strategies to prevent mortality after hip fracture. Approaches that combine both orthopedic and geriatric management have been studied but these have provided conflicting results [16]–[20] and few studies report an improvement in short and long term clinical outcomes [21].

To address this significant public health problem, we formed a multi-disciplinary management team and created a dedicated care unit with the aim of providing integrated postoperative orthopedic and geriatric care for elderly patients with hip fracture. We hypothesized that the provision of early care with specific management of these patients focused on co-morbidity management and rehabilitation would significantly impact long term mortality. To evaluate the impact of this strategy we conducted an interrupted time series study. In addition, we used a national registry to provide external validation of our results.

## Methods

### Ethics statement

Our hospital ethics committee (CPP Ile de France VI, Paris, France) approved this study and authorized waived informed consent since the study was observational. The database was declared to the French National Commission on Computing and Liberty (CNIL, Paris, France).

### Patients

From September 2005 to March 2012 all consecutive patients admitted to our Emergency Department (ED) were evaluated for eligibility. Patients were included if their primary presentation was due to hip fracture and if they were ≥70 years of age. Patients were excluded if they presented with multiple fractures, a metastatic fracture, a fracture complicated by a previous hip prosthesis or osteosynthesis, if they had been transferred to another hospital before surgery, or were already hospitalized at the time of diagnosis.

### Intervention

In June 2009, we created a new geriatric unit (Unit for Post-Operative Geriatric Care, UPOG) devoted to the post-operative care of elderly patients with hip fracture. Before its opening, the medical staff from the emergency, anesthesiology and critical care, geriatric, orthopedic surgery, and rehabilitation departments met to define priorities for patients who would be admitted. Four key factors were identified: (1) early alert from the ED; (2) consideration of hip fracture as an emergency case requiring emergency surgery as soon as feasible (*i.e.* 24 hours a day); (3) rapid transfer to the UPOG after surgery (<48 h); and (4) rapid transfer of stable patients to a dedicated rehabilitation unit. Management strategy focused on early mobilization -with the aim of chair-sitting and walking (first steps) within 24 and 48 hours after arrival respectively, pain management -using acetaminophen and morphine, the provision of air-filled mattresses for patients with pressure ulcers or a high risk of pressure ulcers as evaluated by the Braden scale [22], swallowing disorders detected using a systematic medical survey, detection of stool impaction and urinary retention using ultrasound, the presence of anemia and liberal transfusion of packed red blood cells (usually when the hemoglobin level was <10 g.L^−1^), detection of delirium using the Confusion Assessment Method [23], and malnutrition detection and management in conjunction with a nutritionist. All skills are regrouped in the same ward, allowing a common plan of care for all patients, implicating physicians, nurses, physiotherapists, speech therapist and nutritionist.

### Data collection

Data were collected from computerized ED medical charts (instituted September 1^st^, 2005) and from hand-written medical charts of other departments. Since the opening of the UPOG in June 2009, data were prospectively entered in the database. The following variables were collected: age, sex, home or nursing home living conditions, walking ability, previous medical history, type of fracture and surgical treatment, delay and duration of surgery. Co-morbidity severity was assessed using the Cumulative Illness Rating Scale (CIRS) in which co-occurring medical conditions are weighted from 0 to 4 in 13 main systems [24]. We recorded the preoperative hemoglobin level and its lowest value during the acute care period. Anemia was defined following WHO guidelines [25]. We measured serum creatinine and estimated creatinine clearance [26]. All complications during the acute care period were recorded including delirium, need for physical restraints, stool impaction, urinary retention requiring drainage, morphine administration, pressure ulcer, infection, thromboembolic event, need for blood transfusion, aspiration related to swallowing disorders, cardiac insufficiency (*i.e.* acute cardiac failure or acute pulmonary edema), and admission into an intensive care unit (ICU).

Patients were followed until death or 6 months after admission. Surviving patients or their family were contacted and interviewed by telephone. Missing patients were tracked through health care providers, particularly general practitioners, or any identified acquaintances.

### Study cohort

**Figure 1:**
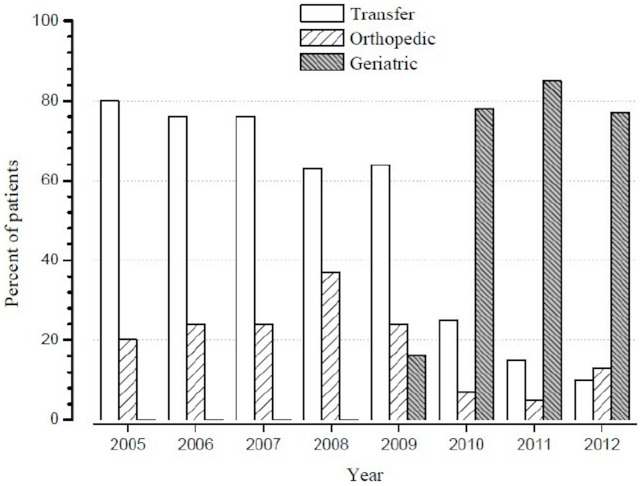
Transfers and allocation of patients. Evolution of transfers out of the hospital (n = 392) and allocation to the orthopedic (n = 131) and geriatric (n = 203) cohorts during the study period. There were only 4 months (September to December) in 2005 and 3 months (January to March) in 2012.

We compared the intervention cohort of patients admitted to the UPOG (*geriatric cohort*) to a control cohort of patients admitted to the orthopedic surgery department (*orthopedic cohort*). All patients transferred >48 h after surgery to the UPOG were assigned to the orthopedic cohort. After the opening of the UPOG, the proportion of patients admitted to the orthopedic department rapidly decreased as did the proportion of transfers to other hospitals (Figure 1), providing a nearly perfect time series analysis [27]. There was no selection of the patients admitted into the UPOG. Nevertheless, before the opening of the UPOG, a selection of patients admitted to the orthopedic department (versus transfer) was very likely. Thus it was expected that the co-morbidities of the cohorts may differ, the patients in the orthopedic cohort being expected to be less severe.

### External validation cohort

We identified all patients admitted to French private or public health institutions in 2010 (n = 7,051,113) (See Table S1 in File S1). To create a hip fracture validation cohort we identified all patients undergoing surgery for hip/femur fracture related to a recent trauma, and then excluded patients <70 years of age and those from our institution. We then extracted patient age, sex, length of acute care stay and presence of pre-existing medical conditions (See Table S2 in File S1) and in-hospital mortality.

### End points

The primary end point was 6-month mortality. The main secondary endpoints were re-hospitalization, re-fracture, new admission into a nursing home, and ability to walk 6 months after admission. We also considered length of stay and mortality while in acute care and rehabilitation facilities, admission into an ICU, delay to first sitting and first walking, and the ability to walk after the acute care and/or rehabilitation period. For the comparison with the external national validation cohort, only hospital mortality was available and this was used as the primary endpoint.

### Sample size calculation

Assuming a baseline 6 month mortality rate of 20% [7] and a mortality reduction of 40% (*i.e.* from 20% to 12%) in the geriatric cohort, we estimated that we would require 298 patients to obtain a 80% power with a two-tailed *P* value of 0·05. This estimation hypothesized a weak relationship between the predictors of the primary endpoint and the strategy tested, which was sustained by the design of the study and by the absence of major change in the recruitment of these patients. A study period of at least 30 months after the opening of the UPOG was planned.

### Statistical analysis

Data are presented as mean ± SD, median [25–75 interquartile] for non-Gaussian variables, or number (percentages). Comparison between cohorts was performed using the unpaired Student t test, Mann-Whitney test, Fisher's exact method, and multivariate analysis of variance when appropriate. Survival was estimated by the Kaplan-Meier method and differences were assessed by the log-rank test. In a preliminary analysis (n = 100) using a multivariate Cox proportional-hazards model we determined that three variables (age, sex, CIRS) were associated with 6-month mortality. We tested the impact of the intervention by calculating the hazard ratio and its 95 percent confidence interval (CI) in association with these prognostic variables.

To provide external validation all patients in the geriatric and orthopedic cohorts were matched with patients drawn from the external national validation cohort. Logistic models using all variables specified in Table S2 in File S1 were developed to determine the probabilities to be in the geriatric or in the orthopedic cohorts. These probabilities (*i.e.* propensity scores) were used to match the patients of the geriatric cohort and those of the orthopedic cohort to patients from the national cohort. Matching was performed using a nearest neighbor matching method with a caliper of 20% of the *logit* of these probabilities. Each patient from the geriatric and orthopedic cohorts was matched to 3 patients from the national cohort using this probability [28]. The absolute standardized difference (ASD) was used to assess balance between the groups. An ASD above 10 to 15% is considered to represent meaningful imbalance [29]. The average treatment effect on the treated (ATT) was estimated in these two cohorts, which represents the average impact of the care program among those who have been exposed to it, was estimated in these two cohorts. We also conducted a sensitivity analysis without caliper use.

All P values were two-sided and P<0.05 was considered significant. R 2.14 software (www.cran.r-project.org last date accessed February 2, 2013) was used for statistical analyses.

## Results

**Figure 2:**
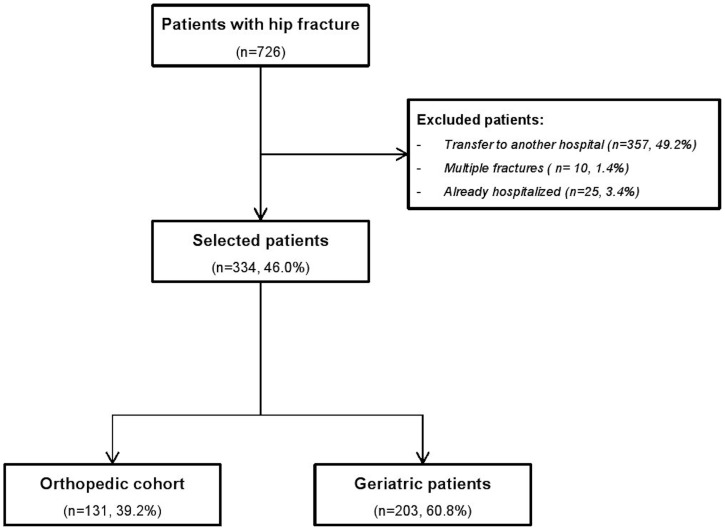
Study flow chart.

Among the 726 elderly patients with hip fracture admitted to the ED, 334 were selected, 131 in the orthopedic cohort and 203 in the geriatric cohort (Figure 2). Only 3 patients were transferred to UPOG more than 48 hours after surgery and so were assigned to the orthopedic cohort.

Patients from the two cohorts differed slightly in the prevalence of co-morbidities and the proportion who received gamma nail and dynamic screw fixation (Table 1). Patients in the geriatric cohort underwent surgery 1 hour before the orthopedic cohort, a clinically insignificant difference but the proportion of patients delayed for >48 h did not. Surgery duration was shorter in the geriatric cohort, probably as a result of the higher proportion of gamma nails (effect of surgical treatment: F = 11.92, *P*<0·001; effect of cohort: F = 0·09, *P* = 0·78) (Table 1). The observed difference in surgery duration (10 min) was not considered to be clinically relevant.

**Table 1:** Comparison of the main characteristics of the two study cohorts.

	Orthopedic cohort	Geriatric cohort	All patients
	(n = 131)	(n = 203)	(n = 334)
Age (years)	85±6	86±6	86±6
Male	44 (34)	50 (25)	94 (28)
**Medical history**			
Obesity^a^	3 (2)	20 (10)	23 (7)
Dementia	32 (24)	78 (38)*	110 (33)
Diabetes	16 (12)	27 (13)	43 (13)
Hypertension	74 (56)	138 (68)*	212 (63)
Cardiac failure	18 (14)	33 (16)	51 (15)
Coronary artery disease	22 (17)	27 (13)	49 (15)
Heart valve disease	9 (7)	17 (8)	26 (8)
Atrial fibrillation	28 (21)	47 (23)	75 (22)
Peripheral vascular disease	5 (4)	12 (6)	17(5)
Stroke	17 (13)	33 (16)	50 (15)
Hemiplegia/paraglegia	1 (1)	8 (4)	9 (3)
Cancer	24 (18)	37 (18)	61 (18)
COPD	10 (8)	15 (7)	25 (7)
Pulmonary hypertension	0 (0)	2 (1)	2(1)
Alcohol abuse	2 (2)	10 (5)	12 (4)
Chronic renal insufficiency	16 (12)	31 (15)	47 (14)
Creatinine clearance (ml.min^−1^)^b^	53±25	53±22	53±23
CIRS 52	5 [3–8]	8 [6–11]	7[4–10]
Hemoglobin (g.dL^−1^)	12.0±1.8	12.1±1.4	12.1±1.5
Anemia	70 (53)	98 (48)	168 (50)
**Living status**			
Living at home	117 (89)	182 (90)	299 (90)
Living in institution	14 (11)	21 (10)	35 (10)
Unknown	0	0	0
Live alone	42 (37)	28 (14)*	70 (21)
Unknown	19	0	19
**Walking ability**			
No walking disability	118 (90)	187 (92)	305 (91)
Moderate walking disability	11 (8)	14 (7)	25 (7)
Does not walk	2 (2)	2 (1)	4 (1)
Unknown	0	0	0
**Fracture**			
Femoral neck fracture	59 (45)	112 (55)	171 (51)
Intertrochanteric fracture	72 (55)	91 (45)	163 (49)
**Surgery**			
Delay to surgery (h)	23 [15–40]	22 [12–34]	22 [14–35]
Delay to surgery >48 h	25 (19)	26 (13)	51 (15)
Duration of surgery (min)	150 [120–175]	140 [110–160]*	140 [120–170]
Gamma nail	33 (25)	102 (50)*	135 (40)
Dynamic hip screw	39 (30)	24 (12)*	63 (19)
Unipolar prosthesis	52 (40)	70 (34)	122 (36)
Bipolar prosthesis	7 (5)	7 (3)	14 (4)

Data are mean ± SD, median [25–75 interquartile], or number (percentage). COPD: chronic obstructive pulmonary disease; CIRS: cumulative illness rating scale;

^a^ : defined as body mass index >30 kg.m^−2^;

^b^ : creatinine clearance could be calculated in 99 (76%) and 200 (98%) patients in the orthopedic and geriatric cohorts respectively.

: P<0.05 vs Orthopedic cohort.

During the acute care period, patients in the geriatric cohort received more morphine, less physical restraint, were transfused more frequently, and were diagnosed with stool impaction and swallowing disorders more frequently. They were less frequently admitted into an ICU and suffered fewer pressure ulcers. However episodes of cardiac insufficiency occurred more frequently. Patients in the geriatric cohort had a markedly reduced time to first sitting and first walking, a much shorter stay in acute care, and a greater proportion of walking patients at the end of the intervention period (Table 2).

**Table 2:** Acute care, rehabilitation, and walking ability.

	Orthopedic cohort	Geriatric cohort	P value
	(n = 131)	(n = 203)	
Delay to first sitting (days)	3 [2–4]	1 [1–2]	<0.001
Delay to first walking (days)	5 [3–9]	2 [1–4]	<0.001
Walking initially contra-indicated	8 (6)	9 (4)	0.61
**Acute care complications**			
Delirium	49/118 (41)	72/203 (35)	0.29
Physical restraint	18/121 (15)	1/203 (0.5)	<0.001
Morphine administration	37/116 (32)	152/203 (75)	<0.001
Swallowing disorders	8/120 (7)	56/203 (28)	<0.001
Lowest hemoglobin (g.dL^−1^)	9.3±1.7	9.2±1.3	0.54
Blood transfusion	72/131 (55)	141/203 (69)	0.008
Stool impaction	23/120 (19)	83/203 (41)	<0.001
Urinary retention	26/120 (22)	57/203 (28)	0.24
Pressure ulcer	40/121 (33)	18/203 (9)	<0.001
Acute heart failure	6/120 (5)	33/203 (16)	0.002
Infection	31/123 (25)	40/203 (20)	0.27
Venous thromboembolism	1/122 (1)	10/203 (5)	0.06
Fall	9/120 (7)	9/203 (4)	0.32
Admission into ICU	17/131 (13)	8/203 (4)	0.005
LOS acute care (days)	13 [10–20]	11 [8–16]	0.001
Admission to rehabilitation care^a^	91/121 (75)	167/197 (85)	0.04
LOS rehabilitation care (days)	41 [25–71]	42 [30–62]	0.78
Total LOS (acute and rehabilitation care) (days)	43 [22–70]	49 [30–68]	0.41
Death during acute care	10/131 (8)	6/203 (3)	0.07
Death during rehabilitation	10/91 (11)	14/166 (8)	0.51
Death during acute care and/or rehabilitation	20/130 (15)	20/202 (10)	0.17
Return to home^b^	92/129 (71)	149/202 (74)	0.70
New admission into nursing home^c^	14/97 (14)	25/163 (15)	1.00
Unknown	2	1	
Readmission within 30 days^d^	19/111 (17)	10/183 (5)	0.002
Redo surgery within 30 days	6/130 (5)	3/201 (1)	0.16
Unknown	2	1	
***Walking ability***			
***After acute care/rehabilitation***			
No walking disability	41/111 (37)	88/182 (48)	0.07
Moderate walking disability	55/111 (50)	88/182 (48)	0.90
Does not walk	15/111 (14)	6/182 (3)	0.002
Missing data	0	1	
***After 6 months***			
No walking disability	33/99 (33)	57/172 (33)	1.00
Moderate walking disability	56/99 (57)	107/172 (62)	0.37
Does not walk	10/99 (10)	8/172 (5)	0.13
Missing data	1	2	
Never walked	29/131 (22)	12/203 (6)	<0.001

Data are mean ± SD, median [25–75 interquartile], or number (percentage). LOS: length of stay; ICU: intensive care unit;

^a^ : excluding death during acute care;

^b^ : institution was considered as “home” in patients previously living in an institution;

^c^ : excluding patients previously living in an institution;

^d^ : excluding patients who died in acute care and/or rehabilitation.

**Figure 3:**
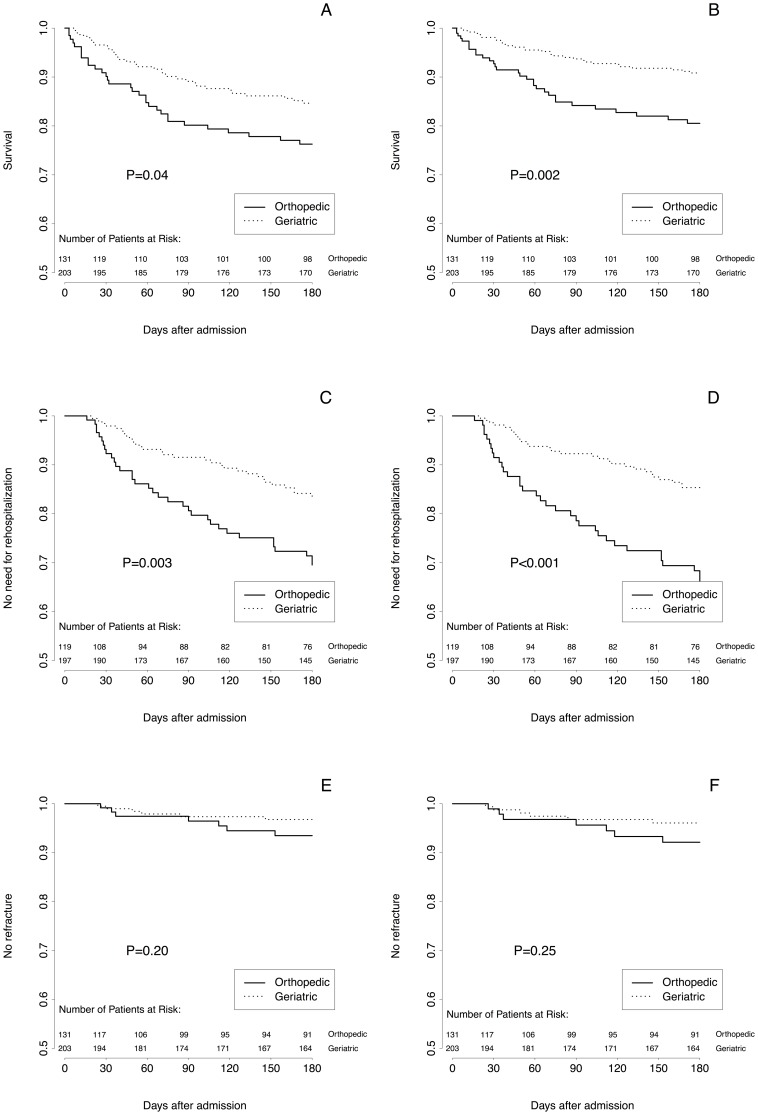
Survival curves for mortality, re-hospitalization, and re-fracture. Survival curves for mortality, re-hospitalization, and re-fracture for patients in the orthopedic (solid lines) and geriatric (dotted line) cohorts. Survival is non-adjusted (panels A, C, and E) and adjusted (panels B, D, and F) for age, sex and Cumulative Illness Rating Scale (CIRS) calculated with a Cox regression analysis. For re-hospitalization and re-fracture, death was considered as a censored observation. *P* values refer to log-rank test.

Only 3 (0.9%) patients were lost to follow-up, 2 in the geriatric cohort and 1 in the orthopedic cohort (all due to transfer to foreign countries). During the follow up period, both mortality and re-hospitalization risks were significantly reduced in the geriatric cohort (Figure 3). When taking into account age, sex, and co-morbidities (using the CIRS), patients in the geriatric cohort showed a profound reduction in both risks of mortality and re-hospitalization. No significant difference was observed for the risk of re-fracture or new admission to a nursing home (Table 3).

**Table 3:** Multivariate cox proportional-hazards analysis predicting death, re-fracture, and re-hospitalization.

Variables	Risk Ratio[95% CI]	P value
**Prediction of death (n = 334)**		
Age	1.04 [1.00–1.08]	0.047
Male sex	1.88 [1.11–3.18]	0.02
CIRS	1.17 [1.10–1.25]	<0.001
Geriatric cohort	0.43 [0.25–0.73]	0.002
**Prediction of re-fracture (n = 334)**		
Age	0.98 [0.90–1.06]	0.58
Male sex	0.22 [0.03–1.76]	0.16
CIRS	1.00 [0.85–1.17]	0.97
Geriatric cohort	0.50 [0.15–1.65]	0.26
**Prediction of re-hospitalization (n = 294)** a		
Age	0.99 [0.95–1.03]	0.68
Male sex	0.76 [0.41–1.41]	0.39
CIRS	1.08 [1.00–1.16]	0.04
Geriatric cohort	0.40 [0.23–0.70]	0.001
**Prediction of admission into a new institution (n = 296)** b		
Age	1.08 [1.03–1.14]	0.003
Male sex	1.71 [0.86–3.41]	0.13
CIRS	1.06 [0.97–1.15]	0.21
Geriatric cohort	0.98 [0.47–2.00]	0.95

CI: confidence interval; CIRS: cumulative illness rating scale;

^a^ : only patients who survived to acute care and rehabilitation were considered;

^b^ : only patients who were not previously living in an institution were considered. For re-hospitalization and re-fracture, death was considered as a censored observation.

**Figure 4:**
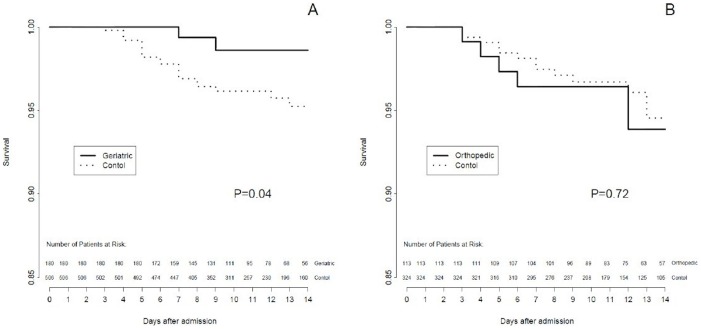
Survival curves for in-hospital mortality. Survival curves for in-hospital mortality in the geriatric (Panel A) and orthopedic (Panel B) cohorts, and their respective matched cohorts from the national registry. *P* values refer to log-rank test.

In the unmatched cohorts, hospital mortality was 7.6 % in the orthopedic cohort (n = 131), 3·0 % in the geriatric cohort (n = 203), and 3.9 % in the external national validation cohort (n = 51,275) but large imbalances were observed between these cohorts (See Table S2 in File S1). After matching, the highest ASD was 9.8% in the geriatric cohort (23 discarded patients) and 13.5% in the orthopedic cohort (18 discarded patients). In the matched populations, hospital mortality was significantly lower in the geriatric cohort compared to the external national validation cohort (2.5% versus 5.4%, log rank test P = 0·04), but not significantly different in the orthopedic cohort (6.3% versus 5.4%, log rank test P = 0.72) (Figure 4). The relative ATT associated to the geriatric cohort indicated a significant reduction in mortality (−63%; 95% CI: −92% to −6%; *P* = 0.006) whereas the relative ATT associated with the orthopedic cohort was not significantly different from zero (32%; 95% CI: −2 to +67%; *P* = 0.57).

In the matching procedure conducted without calipers (*i.e.*, no discarded patients in geriatric and orthopedic cohorts) the population characteristics were not similar with most variables showing an AST>15%. However the relative ATT was −70% (95%CI: −98% to −3%; P<0.001) in the matched geriatric cohort and 51% (95%CI: −12% to 111%; P = 0.83) in the matched orthopedic cohort.

Lastly, we compared the key management factors previously identified (*vide supra*) between patients who were alive at 6 month and those who died. There was no significant difference between groups swallowing disorders (19 vs 26%, P = 0.20), physical restraint (5 vs 10%, P = 0.12), stool impaction (31 vs 39%, P = 0.28), morphine administration (59 vs 62%, P = 0.65), blood transfusion (62 vs 74 %, P = 0.08), and delay for surgery (22 [13–34] vs 23 [15–44] hours, P = 0.23). In contrast, we observed significant differences in pressure ulcers (16 vs 28 %, P = 0.037), delirium (33 vs 61 %, P<0.001), urinary retention (23 vs 37 %, P = 0.04), delay to first sitting (2 [1–3] vs 2 [1–5] days, P = 0.015), delay to first walking (3 [1–5] vs 5 [2–8] days, P<0.001), and proportion of patients who never walked (3 vs 52 %, P<0.001).

## Discussion

In this study, we observed that elderly patients with hip fracture, admitted early into a dedicated geriatric unit and managed with a multidisciplinary approach, had reduced long term mortality and improved walking ability. These observations were externally validated against a matched cohorts derived from a national hospital database.

Various approaches have been taken to integrate orthopedic and geriatric care, also known collectively as orthogeriatrics, for hip fracture patients. As in our study, some of these measures have included admission into a geriatric ward under the specialist care of an orthopedic consultant [16], [17]. However, no previous studies have demonstrated a clear mortality benefit with this approach. Alternative approaches have included using an orthopedic ward with geriatric consultation [30], or an orthopedic ward with daily geriatric management [31]–[33]. When these approaches have shown mortality reduction, it was transient or without reduction in detailed patient morbidity. To our knowledge, this is the first orthogeriatric study that has shown sustained mortality reduction, together with improved walking ability and less morbidity.

We observed a marked reduction (risk ratio 0.43) in the risk of death at 6 months (Table 3). This treatment effect is approximately twice that observed in randomized trials conducted to demonstrate the benefit of early surgery in hip fracture patients [34]. In the geriatric cohort we observed a significant reduction in the length of stay in acute care facilities, but this was not observed when taking into account the rehabilitation period. We believe that some patients in the orthopedic cohort may have been discharged prematurely and that this may at least partially explain the higher proportion of readmitted patients. However, we cannot rule out the possibility that these differences may be due to the lower number of co-morbidities in the orthopedic cohort. When comparing patients who survived at 6 months and those who did not, it seems that early sitting and walking, prevention of pressure ulcer, early identification of urinary retention and delirium, may be the most important management factors associated with survival improvement. Irrespective of the reasons behind this difference our results emphasize the role played by the rehabilitation facilities as well as the importance of the cooperation between acute care and rehabilitation facilities.

Despite considering geriatric hip fracture as an emergency requiring surgery as soon as feasible (i.e. 24 hours a day) [34], [35], we were unable to observe any significant reductions in the time delay until surgery (Table 1). However, in both cohorts, the proportion of patients with delayed surgery was lower than that reported in previous studies [19], [30], [35], suggesting that this goal had already been appropriately implemented in our hospital. Moreover, surgery must sometimes be postponed in some patients due to their pre-existing conditions, including drugs that interfere with hemostasis. The delays to surgery reported in previous orthogeriatric studies were usually longer [19], [30], [35].

Some events in the geriatric cohort such as stool impaction, swallowing disorders, venous thromboembolism, and acute heart failure were more frequently reported. Although these differences may be due to the retrospective recording of data in the orthopedic cohort, we believe that they were detected more frequently in the UPOG due to improved surveillance. Swallowing disorders are a strong risk factor for the development of aspiration pneumonia, and their detection has led to food consistency being thickened and heightened pneumonia surveillance. Despite this we did not observe a significant difference in infection rates. Stool impaction represents a source of discomfort for patients, with increased risk of urinary retention, which may delay rehabilitation and result in life-threatening complications [35]. We cannot rule out the hypothesis that increased morphine consumption in the geriatric cohort may have lead to higher rates of stool impaction. Improved surveillance probably explains the increased venous thromboembolism detection while the increased incidence of acute heart failure could be related either to better clinical detection or intolerance to blood transfusion. However, these differences probably had a limited influence on outcome.

Patients in the geriatric cohort received more blood transfusions and this is explained by our strict adherence to French national transfusion guidelines [36]. These recommend maintaining a hemoglobin >10 g/dL in geriatric patients who are unable to tolerate anemia. This goal was modified in 2011 after Carson et al. [37] showed no benefit of a liberal transfusion regimen as compared to a restrictive one.

Our study has several limitations. First, it was an observational study and the orthopedic cohort data were collected retrospectively. However, we validated our results externally against matched cohorts derived from a national hospital database, used mortality as our primary endpoint, and successfully followed-up 99% of enrolled patients. When comparing complex health procedures, randomized trials are difficult to conduct and interrupted time series analysis are often used. A multicenter cluster randomized study may also be an appropriate methodology. Second, we were unable to compare our results to other models of orthogeriatric reported in the literature [15]–[19]. Comparisons of this nature are difficult due to differences between health care systems and/or local hospital organization, varying study end points, and a lack of reported long term outcomes [30]–[33]. However, our results suggest that an alignment of multidisciplinary hospital teams (physicians and nurses) and hospital care paths (from ED admission to rehabilitation care) toward optimal care of the geriatric hip fracture patient is key factor in successful patient management. Lastly, our results may in part be attributable to certain characteristics of the French health care system and may not be appropriate in countries with different health systems.

## Conclusion

We observed that elderly patients with hip fracture, admitted early into a dedicated geriatric unit and managed with a multidisciplinary approach, had reduced long term mortality and an improved walking ability.

## Supporting Information

File S1 **Contains the following: Table S1. Classification of ICD-10 codes into disease groups. Table S2. Comparison of the main characteristics of the matched populations.**
(DOCX)
