# Supplementary material for: Postoperative Admission to a Dedicated Geriatric Unit Decreases Mortality in Elderly Patients with Hip Fracture
Source: PLoS One. 2014 Jan 15;9(1):e83795. doi: 10.1371/journal.pone.0083795 (PMC3893076; doi:10.1371/journal.pone.0083795)
Supplement: File S1 — Contains the following: Table S1. Classification of ICD-10 codes into disease groups. Table S2. Comparison of the main characteristics of the matched populations. (DOCX) [file pone.0083795.s001.docx]

**Online-Only Supplements**

**Extraction of data from the National database**

We used matched data from the French Technical Agency of Information on Hospitals (Agence Technique de l’Information sur l’Hospitalisation (ATIH), Paris, France) to validate our results.

The agency provided data on all new admissions to all private or public health institutions in France. Using this data we identified all admissions that included a surgical procedure (n= 7,051,113) in 2010.

To create a hip fracture validation cohort, we identified all patients undergoing hip surgery for a hip/femur fracture related to a recent trauma, and then excluded patients < 70 years of age and those from our institution.

Using International Statistical Classification of Diseases and Related Health Problems 10th Revision codes we identified patient age, sex, length of stay in acute care and previous medical history. Pre-existing medical conditions were grouped into broader disease categories to match the variables usually recorded during preoperative clinical assessment. This re-categorization was independently performed by four physicians from different specialties (i.e. nephrology, internal medicine, public health and anesthesiology), all with previous experience in ICD classification. Discrepancies between classifications were resolved by consensus. The most frequent concern was the inability to define the timing of the disease coded with ICD-10. As an example, ICD-10, as used in this registry, is not able to determine if atrial fibrillation (ICD-10: I48.x) was present before surgery, or appeared during or after the surgery. In order to reduce the possibility of bias, we excluded these codes for the final classification (eTable 1).

The type and the severity of the primary diagnosis (*i.e.* the reason of hospital stay) are recorded using the Groupe Homogène de Malades (GHM), a coding system similar to the North American Diagnosis Related Group (DRG). The therapeutic interventions performed are recorded according to the Classification Commune des Actes Medicaux (CCAM).

**Table S1: Classification of ICD-10 codes into disease groups**

| **History of cardiovascular disease** | |
| --- | --- |
| Ischaemic heart disease | I20.x, I25.x, Z95.5 |
| Cardiac arrhythmia | I45.6, I45.9, I49.x, R00.0, R00.1, R00.8, Z45.0, Z95.0 |
| Congestive heart failure | I11.0, I13.0, I13.2, I42.0, I42.5-I42.9, I43.x. |
| Valvular disease | I05.x-I08.x, I34.x-I39.x, Q23.0-Q23.3, Z95.2- Z95.4 |
| Peripheral vascular disease | I71.0, I71.2, I71.4, I71.6, I71.9, I73.1, I73.8, I73.9, I79.0, I79.2, K 55.1, K55.8, K55.9, Z95.8, Z95.9 |
| Hypertension | I10, I11.x, I12.x, I13.x, I15.x |
|  | |
| **History of neurological disease** | |
| Dementia | F00.x, F01.x (excluding F01.0), F02.x, F03.x, G30.x, G31.x. |
| Cerebrovascular disease | G46.x, I69.x. |
| Hemiplegia or paraplegia | G04.1, G11.4, G80.1, G80.2, G81.x, G82.x, G83.0-G83.4, G83.9. |
|  | |
| **History of respiratory disease** | |
| Chronic obstructive pulmonary disease | J43.x, J44.8, J44.9 |
| Pulmonary circulatory disorders | I27.x, I28.8, I28.9 |
|  | |
| **Other** | |
| Renal failure | I12.0, I13.1, N18.1, N18.2, N18.3, N18.4, N18.5, N18.9, N19.x. Z49.0- Z49.2 |
| Diabetes | E10.x (excluding E10.0 and E10.1), E11.x (excluding E11.0 and E11.1), E12.x (excluding E12.0 and E12.1), E13.x (excluding E13.0 and E13.1), E14.x (excluding E14.0 and E14.1) |
| Obesity | E66.x |

I20.x : includes all codes from the I20 category, Q23.0-Q23.3: includes all codes from Q23.0 to Q23.3

**Table S2: Comparison of the main characteristics of the matched populations**

|  | **Study cohort** | | | **Hip fracture national cohort** | | | |
| --- | --- | --- | --- | --- | --- | --- | --- |
|  | **Matched orthopedic cohort** | **Matched geriatric cohort** | **All patients** | **Matched to orthopedic cohort** | **ASD (%)** | **Matched to geriatric cohort** | **ASD (%)** |
|  | (n=113) | (n=180) | (n=51,275) | (n=324) |  | (n=506) |  |
| **Age (years)** | 85 + 6 | 86 + 6 | 85 + 6 | 85 + 6 | 2.6 | 85 + 6 | 4.5 |
| **Male** | 30 (27) | 43 (24) | 11338 (22.1) | 86 (27) | 3.1 | 99 (20) | 9.8 |
| **Medical history** | | | | | | | |
| **Dementia** | 25 (22) | 71 (39) | 9203 (17.9) | 78 (24) | 4.4 | 200 (39) | 0,0 |
| **Diabetes** | 8 (8) | 21 (12) | 5393 (10.5) | 37 (11) | 13.0 | 68 (13) | 5.2 |
| **Hypertension** | 61 (54) | 119 (66) | 18998 (37.1) | 188 (58) | 8.0 | 351 (69) | 7.1 |
| **Cardiac failure** | 13 (12) | 25 (14) | 2927 (5.7) | 38 (12) | 0.9 | 61 (12) | 4.8 |
| **Coronary artery disease** | 18 (12) | 19 (11) | 5476 (10.6) | 46 (14) | 4.7 | 58 (11) | 2.4 |
| **Cardiac valve disease** | 7 (6) | 9 (5) | 1408 (2.8) | 22 (7) | 1.7 | 37 (7) | 8.0 |
| **Peripheral vascular disease** | 2 (2) | 6 (3) | 426 (0.9) | 4 (1) | 3.1 | 13 (3) | 3.5 |
| **Cerebrovascular disease** | 9 (8) | 19 (11) | 984 (2.0) | 22 (7) | 3.1 | 52 (10) | 0.8 |
| **Hemiplegia paraplegia** | 1 (1) | 3 (2) | 532 (1.1) | 7 (2) | 13.5 | 10 (2) | 1.4 |
| **COPD** | 8 (7) | 11 (6) | 1385 (2.7) | 19 (6) | 4.4 | 19 (4) | 8.8 |
| **Pulmonary hypertension** | 0 (0) | 1 (1) | 142 (0.1) | 0 (0) | 0.0 | 4 (1) | 1.9 |
| **Chronic renal insufficiency** | 5 (4) | 24 (13) | 1790 (3.4) | 22 (7) | 7.6 | 64 (13) | 1.8 |

Data are mean + SD, or number (percentage), COPD: chronic obstructive pulmonary disease; ASD: absolute standardized difference. †: defined as body mass index > 30 kg.m^-2^.
